# Commentary on “DeepSeek-R1 and GPT-4 are comparable in a complex diagnostic challenge: a historical control study”

**DOI:** 10.1097/JS9.0000000000003194

**Published:** 2025-08-07

**Authors:** Rui Cheng, Sike He, Yunjin Bai

**Affiliations:** a West China School of Medicine, Sichuan University, Chengdu, China; b Department of Urology, Institute of Urology, West China Hospital, Chengdu, China

*Dear Editor,*

We read with great interest the study by Chan *et al* (2025), “DeepSeek-R1 and GPT-4 are comparable in a complex diagnostic challenge: a historical control study”^[1,2]^. The authors explored the diagnostic capabilities of large language models (LLMs) in complex cases, comparing the emerging open-source model DeepSeek-R1 with the more established proprietary model GPT-4. The study not only found the two models to be comparable in diagnostic abilities and quality, but also demonstrated DeepSeek-R1’s capacity to provide diverse differential diagnoses. These findings provide insights into the medical use of open-source models and support the further development of LLMs in clinical care. However, we would like to raise several methodological concerns and offer some interpretations for the observed results.

The study only used cases from the New England Journal of Medicine clinicopathologic conferences (CPC), which introduces several limitations. First, most of the cases come from a single academic center, Massachusetts General Hospital, in the USA, resulting in geographic and population limitations. Moreover, the cases presented in a structured format may not reflect real-world Electronic Health Records, which are often messy and incomplete. Using cases from multiple international databases and in different formats could improve external validity. In addition, although the study claimed the cases whose publication data were after the LLM training cutoff time, training bias may still exist due to online discussions or data exposure on the internet before publication[3]. Including unpublished cases could help reduce this bias.

The study might benefit from categorizing the cases by disease prevalence and clinical specialty. LLMs may show significantly different performance in diagnosing common versus rare diseases[4]. The study mainly focused on rare cases from CPC, which might obscure potential weaknesses of LLMs in diagnosing more prevalent yet still complex diseases. LLMs’ performance in diagnosis may differ across specialties such as neurology or infectious diseases[5]. Sorting cases by specialty could reveal strengths and limitations of LLMs in different clinical contexts, provide evidence for their deployment in various clinical departments, and guide efforts to enrich their medical

In the results section, the study showed that DeepSeek-R1 and GPT-4 have comparable diagnostic abilities. One possible reason is that both models have similarly high effective capacity, so their performance on reasoning tasks is quite close. Another is that they were both trained with reinforcement learning to strengthen their reasoning ability. However, two models had low accuracy in open-ended diagnostic tasks, indicating they may both have cognitive biases or reasoning limitations. This suggests that current LLMs may not be fully ready for clinical diagnostic applications. Moreover, only 48% of cases received the highest differential quality score, indicating that human review may be necessary following LLMs’ diagnosis, as models may generate nearly correct but not optimal diagnoses.

Interestingly, the study found that DeepSeek-R1 provided more differential diagnoses. This may be because DeepSeek-R1, as a deep-thinking model, was trained to consider a wide range of possibilities, leading to more diverse diagnostic outputs. In the future, this feature could be leveraged by medical institutions to customize the model using their own clinical data, enhancing both diagnostic accuracy and the diversity of differential diagnoses, ultimately enabling real-world deployment[6].

Beyond diagnosis, LLMs have also been explored in other areas such as tumor classification, radiology report summarization, and patient education, showing great potential in medical fields^[7,8]^. However, the accuracy and reliability of LLMs still require further investigation, and legal issues must be addressed before clinical deployment. We call for greater attention to these challenges and continued efforts to ensure that LLMs can be integrated safely and effectively into healthcare.

## Data Availability

Not applicable.
